# Association of *BRCA2* N372H polymorphism with cancer susceptibility: A comprehensive review and meta-analysis

**DOI:** 10.1038/srep06791

**Published:** 2014-10-28

**Authors:** Wen-Qiong Xue, Yong-Qiao He, Jin-Hong Zhu, Jian-Qun Ma, Jing He, Wei-Hua Jia

**Affiliations:** 1 State Key Laboratory of Oncology in South China, Department of Experimental Research, Collaborative Innovation Center for Cancer Medicine, Sun Yat-Sen University Cancer Center, Guangzhou 510060, Guangdong, China; 2 Molecular Epidemiology Laboratory and Laboratory Medicine, Harbin Medical University Cancer Hospital, Harbin 150040, Heilongjiang, China; 3 Department of Thoracic Surgery, Harbin Medical University Cancer Hospital, Harbin 150040, Heilongjiang, China

## Abstract

*BRCA2* gene plays an important role in homologous recombination. Polymorphic variants in this gene has been suggested to confer cancer susceptibility. Numerous studies have investigated association between *BRCA2* N372H polymorphism and risk of several cancers, especially breast cancer. However, the results were inconsistent. We performed a comprehensive meta-analysis to provide a more precise assessment of the association between N372H and cancer risk, following the latest meta-analysis guidelines (PRISMA). Forty six studies involving 36299 cases and 48483 controls were included in our meta-analysis. The crude ORs and the 95% CIs were used to evaluate the strength of the association. The results indicated that the *BRCA2* N372H variant was significantly associated with an increased risk of overall cancer (dominant model: OR = 1.07, 95% CI = 1.01–1.13; recessive model: OR = 1.12, 95% CI = 1.02–1.23). Moreover, stratified analyses by the cancer type and source of control observed significantly increased risk associated with *BRCA2* N372H in subgroups with ovarian cancer, non-Hodgkin lymphoma and population-based controls, but not breast cancer or hospital-based controls. We also found such association among Africans. Overall, the meta-analysis suggested that *BRCA2* N372H may be a cancer susceptibility polymorphism. Well-designed and large-scale studies are needed to substantiate the association between *BRCA2* N372H polymorphism and cancer risk.

Cancer remains one of the leading causes of death worldwide, with approximately 8.2 million cancer-related deaths in 2012 (GLOBOCAN 2012). It has long been recognized that the imbalance between DNA damage and repair plays pivotal role in carcinogenesis. As an example, environmental agents such as mutagenic chemicals and certain types of radiation may induce various DNA alterations, which, if not repaired properly, would cause genetic instability, gene mutations, and chromosomal alterations^1^. Therefore, the host DNA repair systems are important in protecting the genomic integrity against cancer-causing agents.

There are a variety of DNA repair pathways in humans, with each responsible for repairing a certain type of DNA damage^2^. Relatively simple single stranded DNA damages are usually restored via three common mechanisms, base excision repair, nucleotide excision repair and DNA mismatch repair^1^,^2^. In contrast, the repair of double-strand DNA breaks (DSBs), the most severe damage, requires more complex mechanisms including homologous recombination (HR) and non-homologous end-joining (NHEJ) repair pathways, that involve BRCA1, BRCA2, RAD51, etc^3^,^4^. *BRCA2* gene is a well-known cancer susceptibility gene. Its protein product, comprised of 3418 amino acid residuals, has multiple cellular functions in repairing of DSBs by HR^5^,^6^,^7^. One of mechanisms, by which BRCA2 participate in DSB repair is through regulating the intracellular shuttling and function of RAD51, another critical protein in HR^8^. Several lines of evidence demonstrated that BRCA2 protein is essential for DSB repair through HR, but not NHEJ. HR repair of DSBs, not NHEJ, are suppressed in BRCA2-null cells, and consequently abnormality in chromosome structure (i.e., chromosome breaks) would mount up during cell cycle progression^9^. It has been know that mutation in *BRCA2* is related to not only increased breast cancer risk, but also increased risk of the ovary, prostate, pancreas, and male breast, partially due to impaired capacity of repairing DNA DSBs as mentioned above. Accumulating studies have indicated that the polymorphic variants in *BRCA2* gene may also confer genetic susceptibility to cancer because of the alteration in DNA repair capacity.

The N372H is the only common non-synonymous polymorphism in the *BRCA2* gene, resulting in the single amino acid substitution of histidine (His, H) for asparagine (Asn, N)^10^. And the consequential amino acid substitution falls into residues 290–453 of BRCA2, a region mediating interaction between BRCA2 and the histone acetyltransferase P/CAF and transcriptional activation of target genes^11^. Therefore, *BRCA2* N372H polymorphism may influence transcriptional activation function of BRCA2 protein.

Over the past decade, many studies were conducted to evaluate the association between *BRCA2* N372H polymorphism and the risk of cancer, mainly breast cancer. Several studies reported that homozygous carriers of the H allele of *BRCA2* N372H have an increased risk of breast cancer^12^,^13^,^14^, when compared to controls. However, one meta-analyses conducted in 2006 including 12 studies showed no significant association between the polymorphism and breast cancer risk^15^. Qiu et al.^16^ performed an updated pooled analysis of 22 studies in 2010, and confirmed the result. However, Qiu et al. also observed a significant association that was restricted to the population-based studies. These conflicting findings may be ascribed to the relatively small sample size in previous studies. Moreover, no meta-analyses have ever been carried out to investigate the relationship between N372H polymorphism and risks of other types of cancer, such as ovarian cancer. To provide a more precise assessment of the association between N372H and cancer risk, we performed a comprehensive meta-analysis by including the most recent and relevant articles.

## Results

### Study characteristics

**Figure 1:**
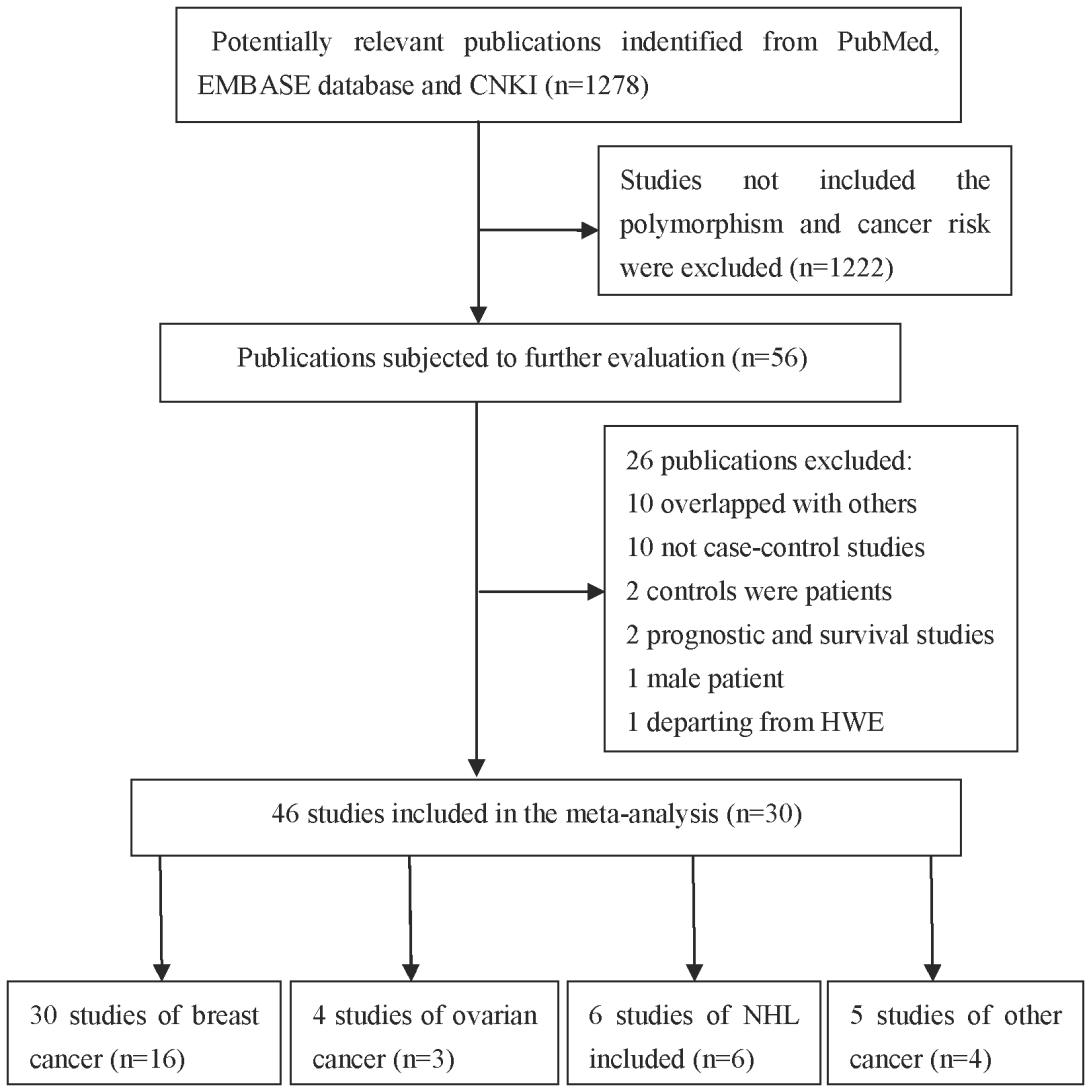
Flow chat of the study screening process in this meta-analysis.

As shown in Figure 1, a total of 1278 relevant articles were retrieved from PubMed, EMBASE and CNKI using search terms described in the methods section. After title and abstract screening, 1222 publications, which did not investigate the association between cancer risk and the polymorphism of interest, were excluded; and then, the remaining 56 publications were carefully reviewed according to the criteria described in the ‘materials and methods' section. Twenty six publications were further removed, among which 10 publications were overlapped with others, 10 were not case-control studies, two used cancer patients as controls, two were prognostic and survival analysis, one was conducted in male patients, and last one departed from HWE. After the removal of all studies that didn't meet our criteria, 46 studies from 30 publications including 36299 cases and 48483 controls were finally included in our meta-analysis (Table 1). Among them, there were 30 breast cancer studies^12^,^13^,^14^,^15^,^17^,^18^,^19^,^20^,^21^,^22^,^23^,^24^,^25^,^26^,^27^,^28^, four ovarian cancer studies^29^,^30^,^31^, six non-Hodgkin lymphoma (NHL) studies^32^,^33^,^34^,^35^,^36^,^37^, two prostate cancer studies extracted from a publication that contained two different populations (Caucasians and African-Americans)^38^ and three other cancer studies^39^,^40^,^41^ (bladder cancer, esophageal squamous cell carcinoma, melanoma). Additionally, one study^42^ combined all the tobacco related cancers together without detailed information for each cancer type. Therefore, this study was only included in our overall analysis, while not in the subgroup analysis by cancer type. Furthermore, 23 studies were considered as low quality (quality score < 9), and 23 were considered as high quality (quality score ≥ 9). All of the cases were histological confirmed, and most controls were matched by sex, age and ethnicity.

### Quantitative synthesis

**Figure 2:**
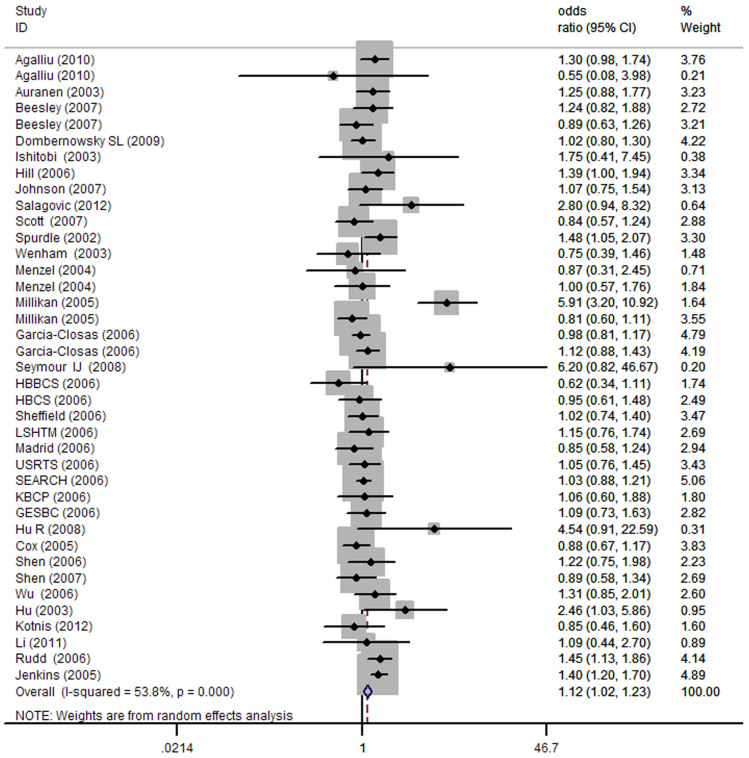
Forest plot of the association between *BRCA2* N372H and cancer risk under homozygous model. For each study, the estimation of OR and its 95% CI are plotted with a box and a horizontal line. 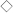, pooled ORs and its 95% CIs.

**Figure 3:**
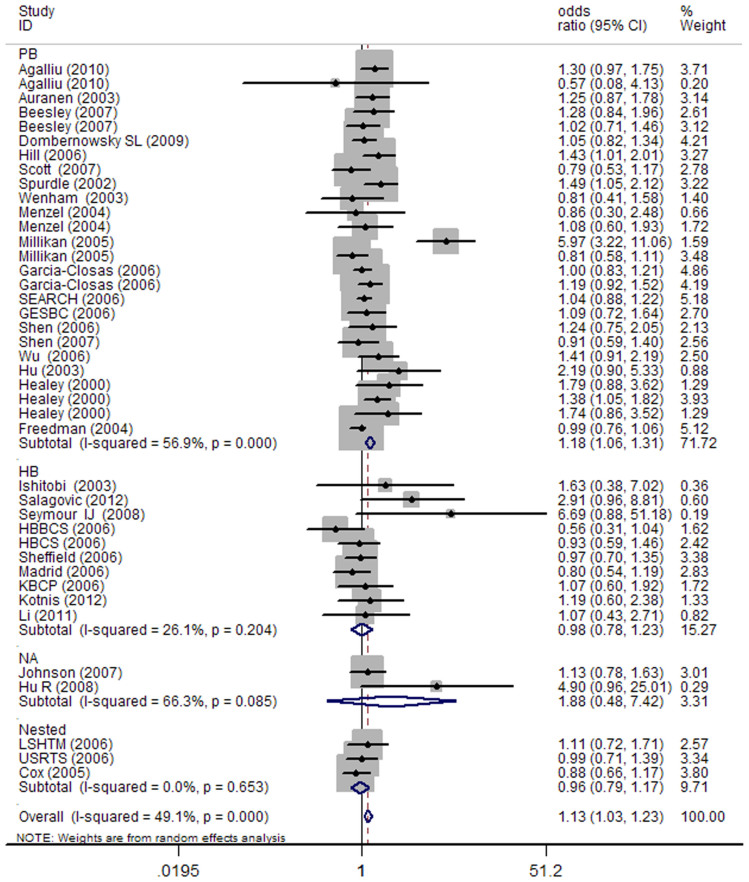
Forest plot of the association between *BRCA2* N372H and cancer risk among control source analysis under homozygous model. For each study, the estimation of OR and its 95% CI are plotted with a box and a horizontal line. 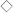, pooled ORs and its 95% CIs.

As indicated in Table 2, pooled analysis yielded a statistically significant association between *BRCA2* N372H variant and overall cancer risk. The overall ORs and 95% CIs among all cancer types were as follows: [homozygous model: OR (95% CI) = 1.13 (1.03–1.23) (Figure 2), dominant model: OR (95% CI) = 1.07 (1.01–1.13); and recessive model: OR (95% CI) = 1.12 (1.02–1.23)]. In the stratified analysis by cancer type, significantly increased cancer risk was found with the minor allele among ovarian cancer [heterozygous: OR (95% CI) = 1.14 (1.02–1.27); dominant: OR (95% CI) = 1.13 (1.02–1.26)] and NHL [recessive: OR (95% CI) = 1.23 (1.01–1.43)] and other cancers [homozygous: OR (95% CI) = 1.37 (1.08–1.73); recessive: OR (95% CI) = 1.35 (1.07, 1.70)]. We further conducted the stratified analysis by ethnicity. As a result, a statistically significant association was found among Africans [dominant: OR (95% CI) = 1.36 (1.10–1.69)] and Mixed group [recessive: OR (95% CI) = 1.33 (1.01, 1.75)]. The stratified analysis by source of controls, observed a statistically significant association in the PB subgroup under all the four models [homozygous model: OR (95% CI) = 1.18 (1.06–1.32); heterozygous model: OR (95% CI) = 1.04 (1.01–1.08); dominant model: OR (95% CI) = 1.11 (1.03–1.19); recessive model: OR (95% CI) = 1.14 (1.01, 1.29)] (Figure 3), but not in the HB subgroup. Finally, when studies were stratified by quality score, a statistically significant association was observed in the high score subgroup under three models [homozygous model: OR (95% CI) = 1.17 (1.03–1.32); dominant model: OR (95% CI) = 1.06 (1.02–1.10); recessive model: OR (95% CI) = 1.09 (1.01, 1.17)], while in low score subgroup, statistically significant association was only observed under recessive model [OR (95% CI) = 1.16 (1.06, 1.26)] (Table 2).

*Q* test and *I^2^* statistic were used to assess the heterogeneity among the studies. There was no heterogeneity under the heterozygous model (*P* = 0.642, *I^2^* = 0.0%) in the overall analysis. In contrast, significant heterogeneity was observed under the homozygous (*P* < 0.001, *I^2^* = 49.1%), dominant (*P* < 0.001, *I^2^* = 61.6%) and recessive model (*P* < 0.001, *I^2^* = 53.8%). Then, we use meta-regression to determine the source of heterogeneity by cancer type, ethnicity and source of controls. As is shown in Table 3, we found that ethnicity contributed to the heterogeneity in the meta-analysis (homozygous model: *P* = 0.017; recessive model: *P* = 0.001), but not cancer type (homozygous model: *P* = 0.459; heterozygous model: *P* = 0.437; dominant model: *P* = 0.250; recessive model: *P* = 0.577), source of controls (homozygous model: *P* = 0.962; heterozygous model: *P* = 0.797; dominant model: *P* = 0.853; recessive model: *P* = 0.971) and quality score (homozygous model: *P* = 0.340; heterozygous model: *P* = 0.936; dominant model: *P* = 0.931; recessive model: *P* = 0.782).

### Sensitivity analysis

We performed leave-one-out sensitivity analysis by excluding a study at a time and recalculating ORs and 95% CIs. There was no substantial change found in the pooled ORs and 95% CIs during the overall analysis, suggesting the stability of our analysis.

### Publication bias

**Figure 4:**
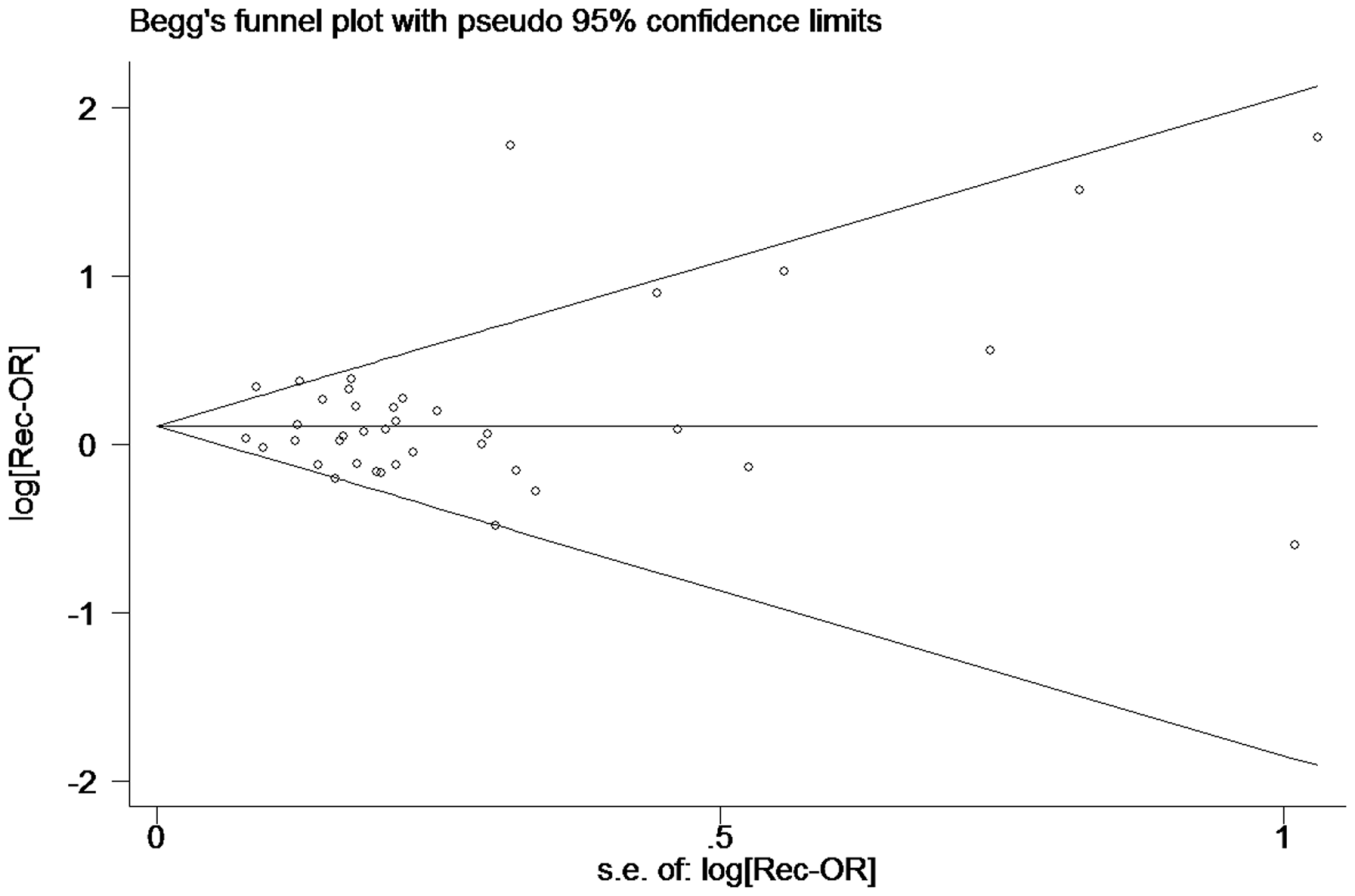
Funnel plot analysis to detect publication bias for *BRCA2* N372H polymorphism by recessive model for overall analysis. Each point represents a separate study for the indicated association.

Begg's funnel plot was used to check the existence of publication bias. There was no evidence of publication bias for the association between polymorphism of *BRCA2* N372H and overall cancer risk under the heterozygous, dominant, or recessive model by using Egger's weighted regression test (*P* = 0.866, *P* = 0.376, *P* = 0.341) (Figure 4), whereas a strong degree of publication bias was found under the homozygous model (*P* = 0.032).

## Discussion

In this comprehensive meta-analysis for the association between *BRCA2* N372H polymorphism and cancer risk, 46 studies with a total of 36299 cases and 48483 controls were included. This great sample size provided adequate statistical power to detect potential association with cancer risk. Pooled analysis suggested a major role of the polymorphism in shaping over cancer risk. Specifically, *BRCA2* N372H polymorphism was shown to confer 13%, 7%, and 13% increases in cancer risk under the homozygous, dominant and recessive models, respectively. Moreover, while stratified analyses were performed by cancer type, significant associations were observed for ovarian cancer, non-Hodgkin lymphoma and other cancers (a combination of prostate cancer, bladder cancer and esophageal cancer), but not for breast cancer. Moreover, stratified analyses by ethnic group and the source of control identified the association among Africans and mixed groups, as well as subgroup with PB controls. To the best of our knowledge, this is the first meta-analysis to evaluate the association between *BRCA2* N372H polymorphism and overall cancer risk.

The *BRCA2* gene is located at chromsome13q13.1 and composed of 27 exons. The BRCA2 protein directly interacts with the RAD51 recombinase to regulate homologous recombination^5^. The N372H polymorphism in the exon 10 is the only identified common non-synonymous polymorphism in *BRCA2* gene, which may confer genetic cancer predisposition. It has been shown that *BRCA2* modulates transcriptional activation of other genes by bonding to histone acetyltransferase P/CAF^11^. *BRCA2* N372H, the non-conservative amino acid substitution-causing polymorphism may influence transcriptional activation function of BRCA2 protein. And a review of epidemiological literatures suggests that this polymorphism may be involved in carcinogenesis^43^. Two meta-analyses have been carried out to attempt to evaluate the association between *BRCA2* N372H and risk of breast cancer. A meta-analysis conducted in 2006 including 15627 cases and 15968 controls revealed no significant association under all the models^15^. With inclusion of more studies, a meta-analysis by Qiu et al. in 2010 also observed no significant association with breast cancer in the overall analysis. Nevertheless, subjects with HH homozygous genotype were reported to have a mild increased risk of breast cancer (OR = 1.11, 95% CI = 1.01–1.21) in PB subgroup^16^. Our results substantiated the previously findings that there was no evidence of association between *BRCA2* N372H and breast cancer risk. However, this polymorphism was shown to significantly increase the risk of ovarian cancer and NHL in stratification analyses by cancer type. Four^29^,^30^,^31^ and six studies^32^,^33^,^34^,^36^,^37^,^44^ on ovarian cancer and non-Hodgkin lymphoma have been conducted before, respectively, but the results were conflicting. The inconsistency may, in part, result from lack of power in individual studies. Moreover, a significant increased cancer risk was also found in PB subgroup, but not in HB or nested case-control subgroup. The null association in HB subgroup is probably due to that controls recruited from hospitals failed to represent the general population from which the cases originated, which may lead to attenuation of risk value.

Moreover, although subgroup analysis by ethnicity found association among Africans, the results should be considered with caution. Since there were only two studies included in the African group, the result may be unstable and false positive, although it was confirmed by meta-regression analysis. Stratification analyses also revealed the between-study heterogeneity observed in the overall analysis might be attributed to the differences among ethnic groups. Many factors may contribute to the strong heterogeneity among overall analysis. The ethnicity-dependent association may be attributed to the discrepancies in genotype distributions among controls of different ethnic groups. Cancer is complicated disease as a result of gene-environment interaction. Therefore different genetic backgrounds among different ethnicities help to explain the ethnicity-dependent results. For example, different populations usually have different linkage disequilibrium types. It is possible that *BRCA2* N372H polymorphism may be in close linkage with another nearby causal variant in one ethnic population but not in another. Moreover, clinical features (e.g., years from illness onset, disease severity) or lifestyle habits (e.g., age, sex, diet) may also explain the heterogeneity of ethnicity. More studies are needed to explore the heterogeneity in the future.

Finally, several limitations in this meta-analysis should be taken into consideration. First, individual studies included in some subgroup analysis, like ovarian cancer and among Africans (<5 studies), may be insufficient. And this could diminish statistical power to detect the potential association. Second, because of lacking the original data from individual studies, our analysis was based on the risk estimates (ORs) without adjustment for other confounding factors. As a result, our findings might suffer from potential confounding bias. Therefore, if possible, future studies should take into account other potential confounding factors to improve the precision of risk estimates for this polymorphism and cancer risk. Third, publication bias also may be one of the concerns because studies with positive results were more prone to be published than those with negative results. The funnel plot and Egger's test suggested that potential publication bias may exist under the homozygous model. Trim-and-fill methods were employed to infer the existence of unpublished hidden studies and yield unbiased pooled estimates. The OR did not significantly change after the publication bias was adjusted (1.13 vs. 1.08). Overall, due to these limitations, the results of this meta-analysis should be interpreted with caution.

In conclusion, this systematical meta-analysis regarding the association between *BRCA2* N372H polymorphism and cancer risk revealed that this polymorphism was significantly associated with an increased risk of overall cancer, and the association was also observed for ovarian cancer. In addition, the significant association was observed in population-based studies while not in hospital-based studies. Finally, well-designed, large-scale studies will be needed to investigate these findings.

## Methods

The meta-analysis was conducted according to the latest meta-analysis guidelines (PRISMA), including literature search, data collection, inclusion, etc.

### Identification of the eligible studies

A comprehensive literature retrieval was performed using the search terms as “*BRCA2* or N372H”, “polymorphism or variation or variant” and “cancer or carcinoma or tumor” through the PubMed and EMBASE up to May 2014 for all relevant studies with no language restriction, according to the latest meta-analysis guidelines (PRISMA)^45^. We also searched the references of the potential relevant publications and bibliographies of the original articles manually in order to find more eligible studies.

### Inclusion criteria

The studies included in our meta-analysis should meet the following criteria: (i) only case-control study or cohort studies were taken into account; (ii) assessed the association between *BRCA2* and risk of cancer; (iii) provided sufficient detail of the genotype frequency or data for estimating odd ratios (ORs) and corresponding 95% confidence intervals (CIs); In addition, studies were excluded if significant departures from Hardy–Weinberg equilibrium (HWE) (*P_HWE_* < 0.05) in controls were observed, unless there were further evidence from other polymorphism of *BRCA2* gene satisfy HWE. When studies had overlapping subjects with others, only the latest or the most complete study was included.

### Data collation

The detailed information was extracted from all the eligible publications independently by two authors as the criteria described above. Conflicts were fully discussed by the two authors until consensuses were reached. The following information from the individual study was collected: name of the first author, year of publication, country of origin, ethnicity, numbers of cases and controls for the *BRCA2* N372H genotypes or the OR and 95% CI under different genetic models, source of controls, genotype method and the *P*-value of HWE in controls. The subgroup analysis was performed by cancer type (cancer subgroups contained less than three individual studies were combined and defined as “others cancer type” group), ethnicity (categorized as Asians, Caucasians, Africans or Mixed which contained more than one ethnic group), the source of controls (HB: hospital-based controls and PB: population-based controls) and quality score (low quality: quality score < 9; high quality: quality score ≥ 9)^46^. We did not define the minimum number of patients for inclusion in our analysis. And publications were divided into different categories or studies when they included different ethnics, cancer types or from different countries.

### Statistical method

The crude ORs and the corresponding 95% CIs were used to evaluate the association between polymorphism of *BRCA2* and cancer risk. For *BRCA2* N372H, the pooled ORs were performed for the homozygous model (CC vs. AA), heterozygous model (CA vs. AA), dominant model (CA + CC vs. AA) and recessive model (CC vs. AA + CA), respectively. Z test was used to determine whether an association was statistically significant. Cochran Q-test and *I*^2^ statistic were used to assess and quantify the between-study heterogeneity (A statistically significant heterogeneity was considered when *P* < 0.10 for Q-test and *I*^2^ represents the proportion of variation in a meta-analysis that is explained by heterogeneity across studies rather than by sampling error.). Fixed-effect model was used if there was no heterogeneity observed when *P* > 0.1 for Q-test; otherwise, a random-effect model was used. Leave-one-out sensitivity analysis was performed by sequentially excluding one single study at a time. Chi-square test was conducted to test if the study is departed from HWE. Additionally, the symmetry of the funnel plot was assessed by Egger's liner regression test to detect the potential publication bias, when at least 4 available dataset were included in subgroup analysis. A meta-regression analysis was performed to investigate the major sources of the heterogeneity across the studies in our meta-analysis.

All statistical tests were performed by STATA version 11.0 (STATA Corporation, College Station, TX), all the *P* values were two-side test and *P* < 0.05 was considered statistically significant.

## Author Contributions

All authors contributed significantly to this work. W.X., Y.H., J.Z. and J.M. performed the research study and collected the data; W.X., Y.H. and J.Z. analyzed the data; W.J. and J.H. designed the research study; W.X., Y.H., J.Z., J.H. and W.J. wrote the paper, and W.X. and Y.H. prepared Figures 1–4, Tables 1–3 and Supplemental Table 1. All authors reviewed the manuscript. In addition, all authors approved the final draft.

## Supplementary Material

Supplementary Information Dataset 1

## Figures and Tables

**Table 1:** Characteristics of studies included in this meta-analysis

First author	Year	Cancer type	Race	Source of control	Case	Control	Method	MAF	HWE
Healey	2000	breast	Caucasian	PB	234	266	TaqMan	/	/
Healey	2000	breast	Caucasian	PB	449	453	TaqMan	/	/
Healey	2000	breast	Caucasian	PB	659	866	TaqMan	/	/
Spurdle	2002	breast	Caucasian	PB	1397	775	Taqman	0.26	0.49
Ishitobi	2003	breast	Asian	HB	149	154	PCR-SSCP	0.20	0.10
Menzel	2004	breast	Caucasian	PB	94	152	Pyrosequencingt	0.26	0.76
Menzel	2004	breast	Caucasian	PB	211	912	Pyrosequencingt	0.27	0.90
Freedman	2004	breast	Mixed	PB	1715	2602	Unknown	/	/
Cox	2005	breast	Caucasian	Nested	1285	1660	Taqman	0.27	0.48
Millikan	2005	breast	African	PB	849	675	Taqman	0.13	0.89
Millikan	2005	breast	Caucasian	PB	1265	1135	Taqman	0.28	0.70
Jenkins	2005	breast	Caucasian	Family	1400	800	Unknown	/	/
HBBCS	2006	breast	Caucasian	HB	274	273	Restriction enzyme-based assays	0.32	0.27
HBCS	2006	breast	Caucasian	HB	807	697	Taqman	0.22	0.40
Sheffield	2006	breast	Caucasian	HB	973	956	Taqman	0.30	0.57
LSHTM	2006	breast	Caucasian	Nested	585	598	Restriction enzyme-based assays	0.29	0.28
Madrid	2006	breast	Caucasian	HB	712	767	Taqman and Illumina	0.30	0.39
USRTS	2006	breast	Caucasian	Nested	707	1046	Taqman	0.30	0.34
SEARCH	2006	breast	Caucasian	PB	4454	4537	Taqman	0.28	0.07
KBCP	2006	breast	Caucasian	HB	446	452	Taqman	0.22	0.61
GESBC	2006	breast	Caucasian	PB	602	851	Various	0.29	0.12
Garcia-Closas	2006	breast	Caucasian	PB	3161	2701	Taqman	0.28	0.09
Garcia-Closas	2006	breast	Caucasian	PB	1968	2276	Taqman	0.26	0.18
Johnson	2007	breast	Caucasian	NA	473	2461	Illumina Sentrix Bead Arrays	0.28	0.88
Seymour	2008	breast	Caucasian	HB	263	60	PCR	0.23	0.13
Hu R	2008	breast	Asian	NA	71	85	PCR	0.22	0.16
Dombernowsky	2009	breast	Caucasian	PB	1200	4119	TaqMan	0.28	0.49
Sun	2009	breast	Asian	PB	512	541	PCR	/	/
Li	2011	breast	Asian	HB	152	165	PCR	0.26	0.56
Silva	2011	breast	Mixed	NA	54	20	PCR	/	/
Auranen	2003	Ovarian	Caucasian	PB	680	1546	TaqMan	0.27	0.12
Wenham	2003	Ovarian	Caucasian	PB	312	398	Taqman	0.25	0.81
Beesley	2007	Ovarian	Caucasian	PB	492	948	MALDI-TOF mass spectrophotometric	0.27	0.38
Beesley	2007	Ovarian	Caucasian	PB	930	825	MALDI-TOF mass spectrophotometric	0.26	0.04
Hill	2006	NHL	Mixed	PB	1116	926	Illumina	0.26	0.67
Shen	2006	NHL	Mixed	PB	476	555	Taqman	0.26	0.46
Scott	2007	NHL	Caucasian	PB	676	757	TaqMan	0.29	0.85
Shen	2007	NHL	Caucasian	PB	556	498	TaqMan	0.30	0.46
Salagovic	2012	NHL	Caucasian	HB	107	127	PCR	0.20	0.97
Rudd	2006	CLL	Caucasian	HB	962	2695	Illumina	/	/
Hu	2003	ESCC	Asian	PB	120	231	PCR–SSCP	0.25	0.13
Wu	2006	bladder	Caucasian	PB	604	595	Taqman	0.25	0.76
Debniak	2008	melanoma	Caucasian	PB + HB	627	3819	RTPCR	/	/
Agalliu	2010	prostate	Caucasian	PB	1269	1243	SNPlex™	0.27	0.62
Agalliu	2010	prostate	African	PB	142	79	SNPlex™	0.14	0.66
Kotnis	2012	overall	Asian	HB	109	186	PCR	0.38	0.01

HB, hospital-based; PB, population-based; MAF, minor allele frequency, HWE, Hardy–Weinberg equilibrium; CLL, chronic lymphocytic leukemia; ESCC, esophageal squamous cell carcinoma.

**Table 2:** Meta-analysis of the association between *BRCA2* polymorphism and cancer risk

Variables	No. of studies^a^	Sample size^b^	Homozygous	P_het_^c^	Heterozygous	P_het_^c^	Dominant	P_het_^c^	Recessive	P_het_^c^
All	46	36299/48483	**1.13 (1.03, 1.23)**	<0.001	1.03 (0.99, 1.06)	0.642	**1.07 (1.01, 1.13)**	<0.001	**1.12 (1.02, 1.23)**	<0.001
Cancer type										
Breast	30	27121/33055	1.11 (0.99, 1.23)	<0.001	1.01 (0.97, 1.05)	0.946	1.05 (0.98, 1.14)	<0.001	1.10 (0.98, 1.24)	<0.001
Ovarian	4	2414/3717	1.13 (0.92, 1.38)	0.587	**1.14 (1.02, 1.27)**	0.154	**1.13 (1.02, 1.26)**	0.384	1.06 (0.87, 1.30)	0.334
NHL	6	3893/5558	1.14 (0.83, 1.57)	0.065	1.01 (0.90, 1.12)	0.547	1.03 (0.92, 1.14)	0.236	**1.23 (1.01, 1.43)**	0.065
Others	5	2762/5967	**1.37 (1.08, 1.73)**	0.580	1.05 (0.95, 1.17)	0.433	1.08 (0.96, 1.22)	0.531	**1.35 (1.07, 1.70)**	0.581
Ethnicity										
African	2	991/754	2.24 (0.23, 21.77)	0.026	1.06 (0.84, 1.33)	0.816	**1.36 (1.10, 1.69)**	0.440	2.19 (0.22, 21.72)	0.025
Asian	6	1113/1362	1.53 (0.99, 2.35)	0.454	1.02 (0.81, 1.27)	0.110	1.33 (0.81, 2.17)	<0.001	1.48 (0.85, 2.59)	0.170
Caucasian	34	30834/42264	1.07 (0.99, 1.16)	0.133	1.02 (0.99, 1.06)	0.605	1.03 (0.99, 1.07)	0.255	1.08 (1.00, 1.16)	0.038
Mixed	4	3361/4103	1.15 (0.90, 1.48)	0.141	1.06 (0.91, 1.23)	0.888	1.06 (0.94, 1.20)	0.681	**1.33 (1.01, 1.75)**	0.660
Source of control										
HB	11	4954/6532	0.98 (0.78, 1.24)	0.204	0.94 (0.86, 1.04)	0.419	0.96 (0.86, 1.07)	0.271	1.07 (0.86, 1.33)	0.063
PB	27	26143/31462	**1.18 (1.06, 1.32)**	<0.001	**1.04 (1.01, 1.08)**	0.746	**1.11 (1.03, 1.19)**	<0.001	**1.14 (1.01, 1.29)**	<0.001
Quality Score										
Low	23	10991/19029	1.06 (0.94, 1.20)	0.288	1.04 (0.98, 1.10)	0.194	1.04 (0.98, 1.10)	0.277	**1.16 (1.06, 1.26)**	0.036
High	23	25308/29454	**1.17 (1.03, 1.32)**	<0.001	1.02 (0.98, 1.06)	0.917	**1.06 (1.02, 1.10)**	<0.001	**1.09 (1.01, 1.17)**	<0.001

^a^, the number of the studies included in our analysis.

^b^, the number of cases and controls included in the studies.

^c^, P value of the Q-test for heterogeneity test.

**Table 3:** Meta-regression analysis of the main characteristics of the 46 studies

Study characteristics	Homozygous			Heterozygous			Dominant			Recessive		
	Coef.	95%CI	*P*	Coef.	95%CI	*P*	Coef.	95%CI	*P*	Coef.	95%CI	*P*
Cancer type	0.02	(−0.04, 0.09)	0.459	0.02	(−0.02, 0.06)	0.437	0.02	(−0.02, 0.06)	0.250	0.01	(−0.04, 0.06)	0.577
Ethnicity	0.20	(0.04, 0.36)	**0.017**	0.02	(−0.11, 0.14)	0.763	−0.01	(−0.09, 0.07)	0.852	0.27	(0.12, 0.43)	**0.001**
Source of controls	−0.003	(−0.13, 0.14)	0.962	−0.01	(−0.09, 0.07)	0.797	−0.01	(−0.09, 0.07)	0.853	0.002	(−0.09, 0.10)	0.971
Quality score	0.04	(−0.24, 0.31)	0.340	−0.01	(−0.17, 0.16)	0.936	−0.01	(−0.17, 0.16)	0.931	0.03	(−0.18, 0.23)	0.782
