# Supplementary material for: Association of BRCA2 N372H polymorphism with cancer susceptibility: A comprehensive review and meta-analysis
Source: Sci Rep. 2014 Oct 28;4:6791. doi: 10.1038/srep06791 (PMC4210867; doi:10.1038/srep06791)
Supplement: Supplementary Information — Dataset 1 [file srep06791-s1.doc]

**Association of** ***BRCA2* N372H polymorphism with cancer susceptibility: A comprehensive review and meta-analysis**

Wen-Qiong Xue ^1^, Yong-Qiao He ^1^, Jin-Hong Zhu ^2^, Jian-Qun Ma^3^, Jing He ^1,*^, Wei-Hua Jia ^1,*^

1 State Key Laboratory of Oncology in South China, Department of Experimental Research, Collaborative Innovation Center for Cancer Medicine, Sun Yat-Sen University Cancer Center, Guangzhou 510060, Guangdong, China

2 Molecular Epidemiology Laboratory and Laboratory Medicine, Harbin Medical University Cancer Hospital, Harbin 150040, Heilongjiang, China

3 Department of Thoracic Surgery, Harbin Medical University Cancer Hospital, Harbin 150040, Heilongjiang, China

Wen-Qiong Xue and Yong-Qiao He contributed equally.

***Correspondence to**: Jing He, State Key Laboratory of Oncology in South China, Department of Experimental Research, Collaborative Innovation Center for Cancer Medicine, Sun Yat-Sen University Cancer Center, 651 Dongfeng Road East, Guangzhou 510060, Guangdong, China. Tel./Fax: (+86-20) 87342410, E-mail: [hejing198374@gmail.com](mailto:hejing198374@gmail.com) or Wei-Hua Jia, State Key Laboratory of Oncology in South China, Department of Experimental Research, Collaborative Innovation Center for Cancer Medicine, Sun Yat-Sen University Cancer Center, 651 Dongfeng Road East, Guangzhou 510060, Guangdong, China, Tel.: (+86-20) 87342327; Fax: (+86-20) 87343392, E-mail: [jiaweih@mail.sysu.edu.cn](mailto:jiaweih@mail.sysu.edu.cn).

| Supplemental Table 1. Score of Quality Assessment | |
| --- | --- |
| **Criteria** | **Score** |
| Representativeness of cases |  |
| Selected from population cancer registry | 2 |
| Selected from hospital | 1 |
| No method of selection described | 0 |
| Representativeness of controls |  |
| Population-based | 3 |
| Blood donors | 2 |
| Hospital-based | 1 |
| Not described | 0 |
| Ascertainment of cancer cases |  |
| Histopathologic confirmation | 2 |
| by patient medical record | 1 |
| Not described | 0 |
| Control selection |  |
| Controls matched with cases by age and sex | 2 |
| Controls matched with cases only by age or by sex | 1 |
| Not matched or not descried | 0 |
| Genotyping examination |  |
| Genotyping done blindly and quality control | 2 |
| Only genotyping done blindly or quality control | 1 |
| Unblinded and without quality control | 0 |
| Total sample size |  |
| Larger than 1000 | 3 |
| Larger than 500, but less than 1000 | 2 |
| Larger than 200, but less than 500 | 1 |
| Less than 200 | 0 |
